# Estradiol trajectories and early pregnancy loss: a retrospective study

**DOI:** 10.3389/fendo.2025.1657453

**Published:** 2025-11-17

**Authors:** Yanling Wei, Xue Wei, Fangxiang Mu, Fang Wang

**Affiliations:** Department of Reproductive Medicine, Lanzhou University Second Hospital, Lanzhou, China

**Keywords:** early pregnancy estradiol, trajectory patterns, early miscarriage, natural conception, risk assessment

## Abstract

**Objective:**

To investigate the association between early pregnancy estradiol (E2) trajectory patterns and the risk of early miscarriage in women with natural conception.

**Methods:**

This retrospective study included 527 women aged 18–45 years with natural conception and at least three E2 measurements within the first 12 gestational weeks, from March 2023 to August 2024. Group-based trajectory modeling identified four distinct E2 trajectories. Demographic and clinical data were extracted from medical records, and pregnancy outcomes were obtained through follow-up. Multivariate logistic regression and subgroup analyses were performed to evaluate the association between E2 trajectories and early miscarriage.

**Results:**

Among the four identified E2 trajectories, women in Trajectory 3 (“High Level with Steady Increase”) showed a significantly reduced risk of early miscarriage compared to those in Trajectory 2 (“Low Level with Slow Increase”) (adjusted OR = 0.24, 95% CI: 0.12–0.46, p < 0.001). Subgroup analyses stratified by age and number of previous miscarriages confirmed the robustness of this association, while no significant associations were found for the other trajectories. The highest miscarriage rate (42.03%) and lowest baseline E2 level (300.29 ± 194.23) were observed in Trajectory 2.

**Conclusion:**

A steadily increasing high estradiol trajectory in early pregnancy is associated with a lower risk of early miscarriage, highlighting the potential value of E2 monitoring for early pregnancy risk assessment.

## 1 Introduction

Early pregnancy loss, especially miscarriage occurring within 12 weeks of gestation (1), is the most common form of pregnancy failure and severely impacts women’s reproductive health and psychological well-being. Its etiology is complex and not yet fully understood (2, 3). In recent years, with the continuous advancement of assisted reproductive technologies and natural pregnancy management, there has been widespread attention on the prevention and intervention of early pregnancy failure (4, 5). Estrogen hormones, particularly estradiol (E2), play a critical role in maintaining early pregnancy and embryonic development (6, 7). E2 not only directly affects the functional remodeling of the endometrium (8) to facilitate embryo implantation (9) but also participates in regulating the bioactivity of progesterone (10) to ensure a proper developmental environment for the embryo (11).

Although prior studies have found that serum E2 levels are generally lower in patients with miscarriage (12, 13), suggesting that E2 levels may be closely related to pregnancy outcomes, these studies mainly rely on static measurements at single or limited time points, which fail to reflect the dynamic trends of estradiol variation, thereby limiting the accuracy of predicting pregnancy outcomes. Estradiol levels during early pregnancy exhibit nonlinear changes and significant individual variability; thus, a single time-point measurement cannot fully reveal its comprehensive effects on the pregnancy process. Therefore, elucidating the specific trajectories of E2 changes in early pregnancy and their associations with miscarriage risk is crucial for early risk assessment and clinical intervention.

In recent years, group-based trajectory modeling (GBTM) has been applied in biomedical research to identify disease progression patterns and hormonal variation trends among potential subtypes (14–16). Compared with traditional cross-sectional analyses, GBTM can accurately capture diverse temporal change patterns within different subgroups, providing a powerful tool for studies in personalized medicine (17). Applying this method to analyze dynamic changes of estradiol during early pregnancy can not only reveal the dynamic structure of hormone levels but also clarify the relationship between different trajectory types and pregnancy outcomes, facilitating the identification of potential high-risk subgroups and optimization of clinical management protocols.

This study is based on the clinical retrospective data of naturally conceived patients from the Department of Reproductive Medicine, Lanzhou University Second Hospital. We established a dynamic trajectory model of estradiol changes measured at multiple points within the first 12 weeks of pregnancy. Using GBTM, we analyzed serum E2 level patterns in 527 patients and explored the associations between different trajectory groups and the risk of early pregnancy miscarriage.

## 2 Methods

### 2.1 Study population

**Figure 1:**
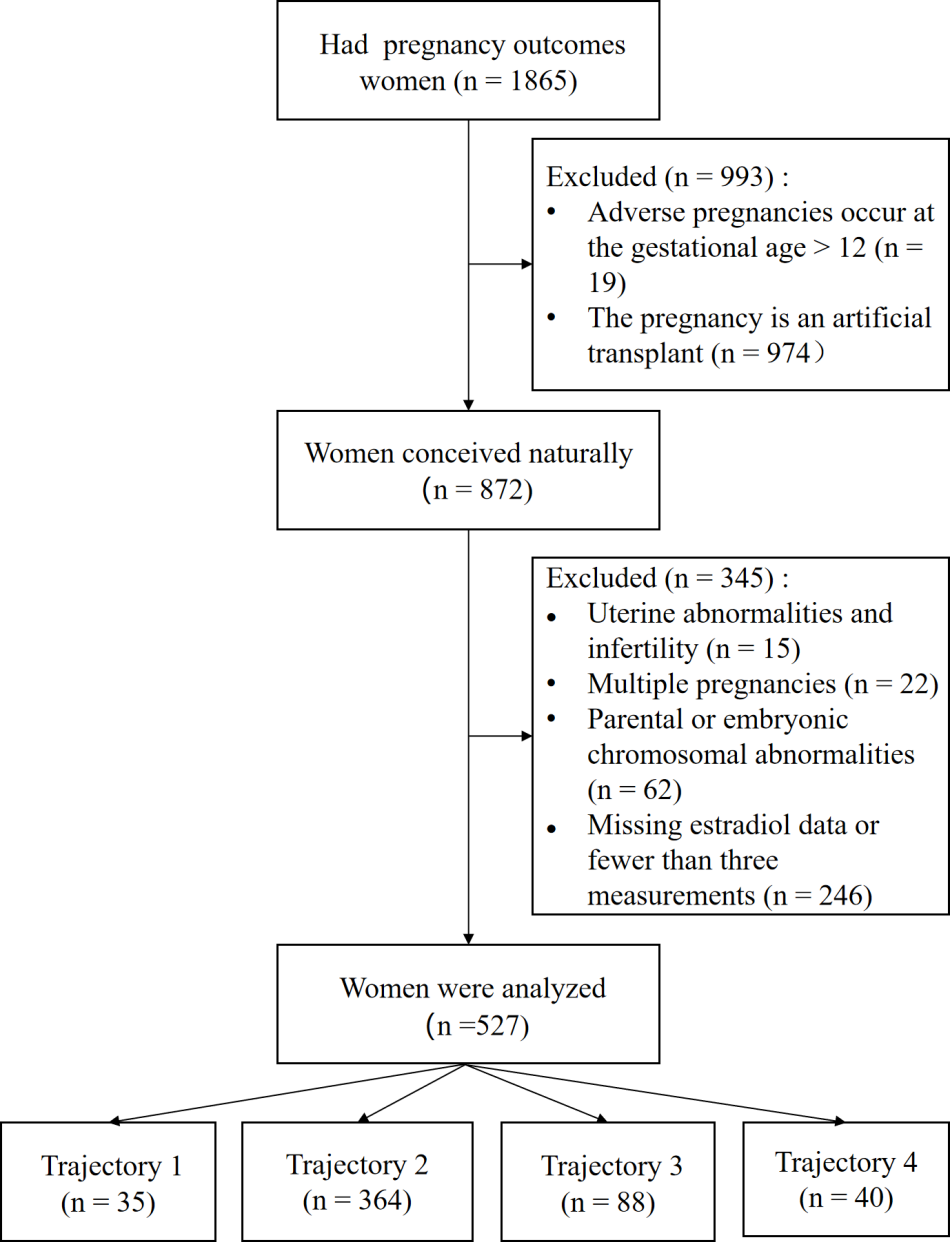
Flow chart.

This retrospective study included 1,865 outpatients who visited the Reproductive Center of the Department of Reproductive Medicine, Lanzhou University Second Hospital between March 2023 and August 2024. The inclusion criteria were (1): age between 18 and 45 years; and (2) natural conception. The exclusion criteria were as follows (1): chromosomal abnormalities in either spouse or embryo (2); congenital uterine malformations (such as septate uterus, unicornuate uterus, bicornuate uterus, or uterus didelphys) (3); multiple pregnancies; and (4) fewer than three estradiol measurements within the first 12 weeks of pregnancy. Ultimately, 527 patients meeting the criteria were included and categorized into four groups based on estradiol level trajectories: Group 1 (n=35), Group 2 (n=364), Group 3 (n=88), and Group 4 (n=40) (see **Figure 1**).

### 2.2 Data collection

Demographic variables were extracted from medical records and included maternal age, weight, height, age at menarche, menstrual regularity, and history of previous pregnancy loss. Body mass index (BMI) was calculated by dividing weight in kilograms by height in meters squared. The number of previous miscarriages was categorized into three groups: one, two, and three or more. Pregnancy outcomes were obtained through follow-up or review of hospital medical records.

### 2.3 Primary outcomes

Early pregnancy loss was defined as miscarriage occurring before 12 weeks of gestation, including biochemical pregnancies. Ongoing pregnancy was defined as continuation of pregnancy at 12 weeks gestation or later. Gestational age was calculated in weeks from the date of conception to the date of the outcome event.

### 2.4 Estradiol measurement

E2 levels were measured using a commercial automated electrochemiluminescence immunoassay system (DxI 800 Immunoassay System, Beckman Coulter, USA). All assays were performed by experienced technicians following the manufacturer’s instructions precisely.

### 2.5 Statistical analysis

Sample size was estimated for a multiple logistic regression analysis. We aimed for 90% power to detect an OR of 0.24 at a two-sided α level of 0.05. The assumptions included a baseline event rate (pregnancy loss) of 42.03%, a 16.7% sample proportion for Trajectory Group 3, and an R-squared of 0.3 from other covariates. Based on these parameters, the calculation using PASS software yielded a required sample size of 286 participants. As our final cohort consisted of 527 patients, the study was adequately powered.

E2 level trajectories were modeled for the 527 patients using Group-Based Trajectory Modeling (GBTM) to identify latent subgroups with similar patterns of E2 changes. Trajectory shapes were explored using linear, quadratic, and cubic polynomial forms to capture different nonlinear trends. Gestational week was used as the time scale, and the number of trajectory groups was varied progressively from two to five. For each number of groups, models were initialized with parameters derived from the one-group model and run multiple times with random starting values to avoid convergence to local maxima.

The best-fitting model was selected based on the following criteria (1): minimum values of Akaike Information Criterion (AIC) and Bayesian Information Criterion (BIC) (2); average posterior probability (APP) greater than 0.70 for each group; and (3) minimum sample size of 5% in any trajectory group. Ultimately, a cubic polynomial model with four trajectory classes was chosen as the optimal fit. Model construction was performed using the “lcmm” package in R version 4.3.1.

Descriptive statistical analyses were conducted for the trajectory groups. Continuous variables were compared using the Kruskal-Wallis test or Student’s t-test, while categorical variables were assessed by Chi-square test or Fisher’s exact test and presented as counts (percentages). Further, multivariate logistic regression models were used to evaluate the association between E2 trajectory groups and pregnancy outcomes: Model 1 was unadjusted; Model 2 adjusted for age; Model 3 further adjusted for age, BMI, number of E2 measurements, and history of previous miscarriage. Subgroup analyses stratified by age and number of previous miscarriages were conducted to assess the consistency of these associations across different population characteristics.

All statistical analyses were performed using R software (version 4.3.1) and EmpowerStats (version 4.2). All hypothesis tests were two-sided, with a significance threshold set at *p* < 0.05.

## 3 Results

### 3.1 Baseline characteristics

The baseline characteristics of the 527 participants are presented in **Table 1**. All participants had at least three estradiol tests during early pregnancy. The cohort comprised 335 women with a 12-week ongoing pregnancy and 192 women with early pregnancy loss. The overall mean age and mean age at menarche were 30.92 and 13.02 years, respectively. The two groups differed significantly in age, BMI, total number of measurements, and history of miscarriage (*p*< 0.05). All other baseline characteristics were comparable between the groups.

**Table 1:** Baseline characteristics of participant.

Characteristics	Total	Ongoing pregnancy	Pregnancy loss	*P*-value
N	527	335	192	
Age, years,mean ± SD	30.92 ± 3.72	30.54 ± 3.64	31.58 ± 3.77	**0.002**
Basic estradiol, pg/ml, mean ± SD	408.40 ± 378.61	408.62 ± 394.23	408.01 ± 350.70	0.986
BMI, kg/m2, mean ± SD	21.82 ± 3.01	21.57 ± 2.95	22.27 ± 3.07	**0.010**
Age at menarche, years, mean ± SD	13.02 ± 1.27	13.05 ± 1.27	12.97 ± 1.27	0.492
Count total occurrences, mean ± SD	8.07 ± 3.65	8.93 ± 3.59	6.57 ± 3.23	**<0.001**
Regularity of menstruation, n (%)				0.584
No	122 (23.15%)	75 (22.39%)	47 (24.48%)	
Yes	405 (76.85%)	260 (77.61%)	145 (75.52%)	
Previous miscarriage				**0.001**
1:1	199 (37.76%)	128 (38.21%)	71 (36.98%)	
2:2	205 (38.90%)	145 (43.28%)	60 (31.25%)	
3:≥3	123 (23.34%)	62 (18.51%)	61 (31.77%)	

SD, standard deviation; BMI, body mass index.Bold values indicate statistical significance.

### 3.2 Identification of number of trajectories

**Supplementary Table 1** summarizes the model fitting process for group-based trajectories with 1 to 5 latent classes using linear, quadratic, and cubic polynomial forms. Although the cubic model with five latent groups had the lowest Bayesian Information Criterion (BIC), one trajectory group included less than 5% of the sample. Therefore, the cubic model with four latent groups was selected as the optimal Group-Based Trajectory Model (GBTM). **Supplementary Table 2** presents the comprehensive parameter estimates for the best-fitting four-class cubic trajectory model.

**Figure 2:**
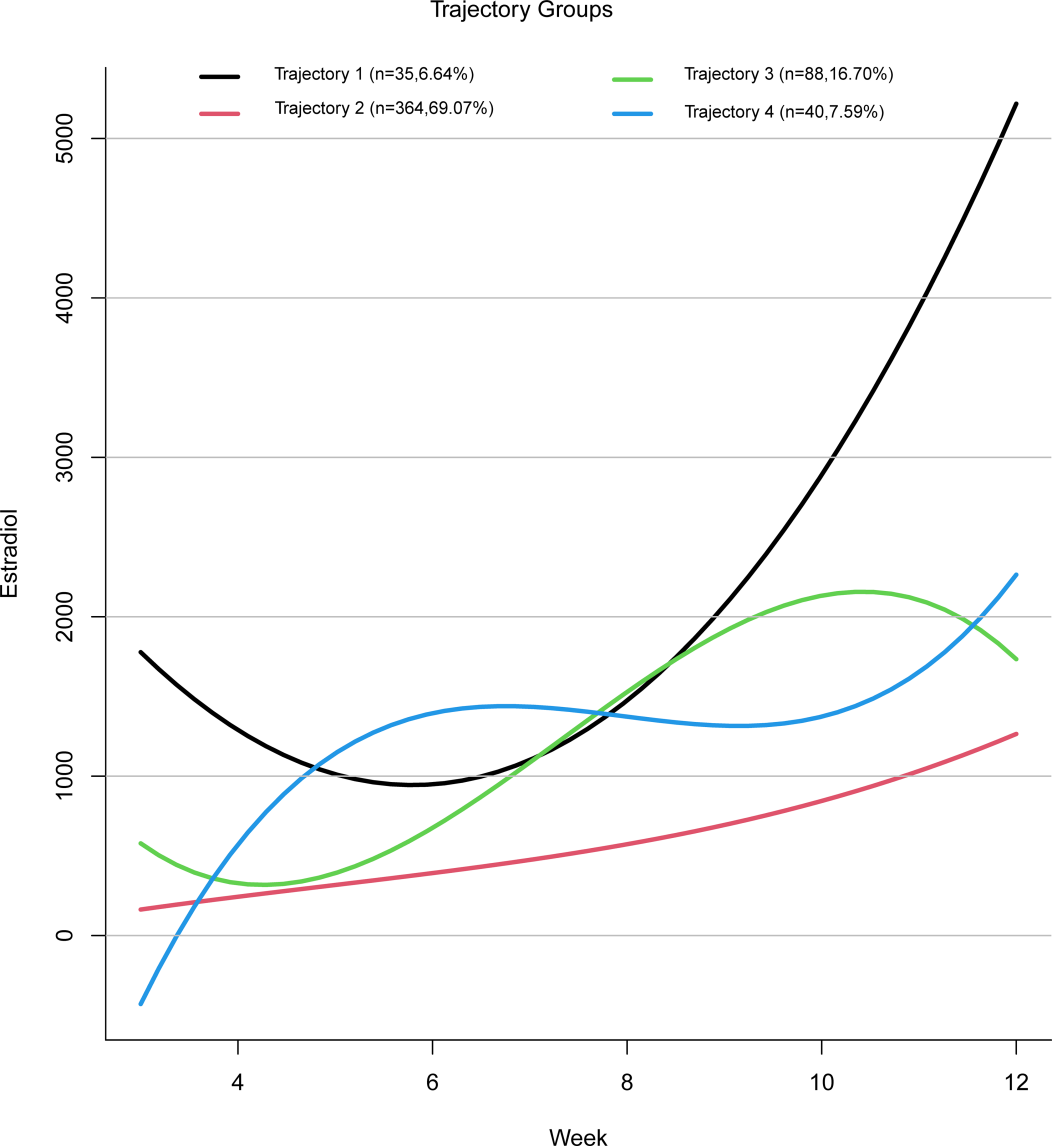
Mean trajectories of estradiol increasing.

Based on the four estradiol trajectories shown in **Figure 2**, participants were classified into four groups:

Trajectory 1: “High Level with Sharp Increase” — characterized by a relatively high initial estradiol level, followed by a decline, and then a rapid increase in later stages.

Trajectory 2: “Low Level with Slow Increase” — characterized by consistently low estradiol levels with a gradual upward trend.

Trajectory 3: “High Level with Steady Increase” — characterized by an initial decline in estradiol levels followed by a faster rise reaching a relatively high level.

Trajectory 4: “Fluctuating Increase” — characterized by an estradiol level that rises initially, then falls, and rises again.

### 3.3 Characteristics of trajectory subgroups

**Table 2** presents the baseline characteristics of participants in each trajectory group. A total of 4,255 estradiol measurements were included in the study, distributed as follows: 259 measurements in Trajectory 1, 2,927 in Trajectory 2, 749 in Trajectory 3, and 320 in Trajectory 4. The four trajectory groups were similar in terms of age, BMI, age at menarche, number of measurements, and menstrual regularity. However, significant differences were observed among the groups in baseline estradiol levels and pregnancy outcomes. Compared with Trajectories 1, 3, and 4, Trajectory 2 showed the highest miscarriage rate (42.03%) and the lowest average baseline estradiol level (300.29 ± 194.23).

**Table 2:** Baseline characteristics of the total sample and the sample by the different trajectory groups.

Characteristics	Total (n = 527)	Trajectory 1 (n = 35)	Trajectory 2 (n = 364)	Trajectory 3 (n = 88)	Trajectory 4 (n = 40)	*P*-value
Total No.^b^	4255	259	2927	749	320	
Maternal age (years), mean ± SD	30.92 ± 3.72	31.89 ± 3.49	30.93 ± 3.70	30.76 ± 3.75	30.35 ± 3.93	0.326
Initial E2 (pg/ml), mean ± SD	408.40 ± 378.61	1036.25 ± 634.28	300.29 ± 194.23	422.76 ± 368.30	811.29 ± 546.90	**<0.001**
BMI (kg/m^2^), mean ± SD	21.82 ± 3.01	22.01 ± 3.56	21.75 ± 2.99	22.31 ± 3.10	21.29 ± 2.39	0.272
Age at menarche (years), mean ± SD	13.02 ± 1.27	12.49 ± 1.12	13.00 ± 1.24	13.18 ± 1.24	13.32 ± 1.59	**0.018**
Total E2 assay, mean ± SD	8.07 ± 3.65	7.40 ± 2.33	8.04 ± 3.81	8.51 ± 3.44	8.00 ± 3.44	0.475
Regularity of menstruation, n (%)						0.076
Irregular	122 (23.15%)	13 (37.14%)	84 (23.08%)	14 (15.91%)	11 (27.50%)	
Regular	405 (76.85%)	22 (62.86%)	280 (76.92%)	74 (84.09%)	29 (72.50%)	
Number of previous pregnancy losses, n (%)						0.430
1	199 (37.76%)	10 (28.57%)	141 (38.74%)	37 (42.05%)	11 (27.50%)	
2	205 (38.90%)	14 (40.00%)	140 (38.46%)	35 (39.77%)	16 (40.00%)	
≥3	123 (23.34%)	11 (31.43%)	83 (22.80%)	16 (18.18%)	13 (32.50%)	
Pregnancy outcomes, n (%)						**<0.001**
Ongoing pregnancy	335 (63.57%)	21 (60.00%)	211 (57.97%)	74 (84.09%)	29 (72.50%)	
Pregnancy loss	192 (36.43%)	14 (40.00%)	153 (42.03%)	14 (15.91%)	11 (27.50%)	

SD, standard deviation; BMI, body mass index.

^b^ Total number of estradiol assay. Bold values indicate statistical significance.

### 3.4 Association between estradiol trajectory groups and pregnancy outcomes

In the unadjusted Model 1, estradiol levels in Trajectory 3 were negatively associated with early miscarriage (OR = 0.26, 95% CI: 0.14–0.48, *p* < 0.001). This association remained significant after adjusting for age in Model 2 (OR = 0.26, 95% CI: 0.14–0.48, *p* < 0.001). In Model 3, which further adjusted for age, BMI, number of measurements, and number of previous miscarriages, no significant association was found between early miscarriage and Estradiol Trajectory 1 (OR = 0.74, 95% CI: 0.35–1.58, *p* = 0.440) or Trajectory 4 (OR = 0.49, 95% CI: 0.22–1.07, *p* = 0.072). However, the negative association between Trajectory 3 and early miscarriage remained significant, with the risk of miscarriage in Trajectory 3 being 0.24 times that of Trajectory 2 (OR = 0.24, 95% CI: 0.12–0.46, *p* < 0.001) (see **Table 3**).

**Table 3:** The association of different trajectory with early pregnancy loss.

Trajectory	Model 1 OR (95% CI)	*P* value	Model 2 OR (95% CI)	*P* value	Model 3 OR (95% CI)	*P* value
Trajectory 2	ref		ref		ref	
Trajectory 1	0.92 (0.45, 1.87)	0.816	0.85 (0.42, 1.75)	0.667	0.74 (0.35, 1.58)	0.440
Trajectory 3	0.26 (0.14, 0.48)	<0.001^*^	0.26 (0.14, 0.48)	<0.001^*^	0.24 (0.12, 0.46)	<0.001^*^
Trajectory 4	0.52 (0.25, 1.08)	0.080	0.54 (0.26, 1.12)	0.098	0.49 (0.22, 1.07)	0.072

OR, odds ratio; CI, confidence interval.

Asterisks indicate p values *P≤ 0.05.Model 1: no adjusted.Model 2: adjusted for age.Model 3: adjusted for age, body mass index, total E2 assay, and number of previous pregnancy losses.

### 3.5 Subgroup analysis

To assess whether the association between estradiol trajectories and pregnancy outcomes was consistent across different population characteristics, subgroup analyses were conducted stratified by age and number of previous miscarriages. Among participants aged 20–29 and 30–44 years, estradiol levels in Trajectory 3 were significantly negatively associated with early miscarriage (*p* < 0.05). Similarly, in participants with one or two previous miscarriages, Trajectory 3 estradiol levels showed a significant negative association with early miscarriage (*p* < 0.05), whereas other trajectory groups did not show significant associations. The subgroup analyses indicated no significant interaction effects of age or history of miscarriage on the relationship between estradiol trajectories and early miscarriage (*p* for interaction > 0.05) (see **Supplementary Table 3**).

## 4 Discussion

In this study of early pregnancies, we identified distinct patterns of serum E2 rise and found that they were associated with miscarriage risk. The trajectory characterized by high E2 levels with a steady rise (Trajectory 3) was associated with the lowest early miscarriage rate, whereas the low-level, slowly rising trajectory (Trajectory 2) carried the highest loss rate. This finding is novel in stratifying risk by dynamic endocrine profiles, and it underlines the critical importance of a robust estrogenic environment in early gestation.

### 4.1 High-level, steadily rising estradiol and low miscarriage risk

Women in Trajectory 3 – those with high initial estradiol and a sustained rise – had very favorable outcomes. This profile likely reflects an optimally functioning corpus luteum (CL) and timely luteal–placental transition. Early pregnancy estradiol is initially produced exclusively by the CL (18) and adequate levels indicate strong ovarian steroidogenic capacity (19, 20). A plentiful estradiol supply would also maintain progesterone secretion and placental growth factors, ensuring a healthy endocrine milieu (21, 22). In fact, predictive modeling in a separate Danish cohort showed estradiol to be the strongest serum predictor of viability, even outperforming hCG and progesterone (23). Taken together, these observations suggest that Trajectory 3 pregnancies experience the “normal” physiologic cascade of endometrial preparation and placentation, thereby minimizing loss.

Estradiol acts on the endometrium to promote proliferation and prepare for the secretory phase (24). In the proliferative (pre-implantation) phase, estrogen induces mucosal growth and upregulates progesterone receptor expressionpmc (24). High, rising estradiol therefore primes the endometrium to respond fully to progesterone, a prerequisite for decidualization and implantation. Parisi et al. emphasize that even during the luteal phase, adequate estrogens are required to activate paracrine signaling for endometrial receptivity (25). By ensuring a thick, well-vascularized lining and sufficient progesterone sensitivity, Trajectory 3 pregnancies likely achieve optimal conditions for embryo embedding.

Furthermore, robust estradiol supports early placental angiogenesis and immune adaptation. Estrogen modulates angiogenic factor expression and helps remodel uterine natural killer (uNK) and T-helper cell function. Thus, a strong estradiol trajectory may foster a healthy, immune-tolerant microenvironment at the maternal-fetal interface (25). Altogether, the data indicate that Trajectory 3 represents a physiologically optimal endocrine pattern, and this aligns with the low observed miscarriage rate in this group.

### 4.2 Low-level, slowly rising estradiol and high miscarriage risk

In stark contrast, pregnancies following Trajectory 2 (low initial estradiol with a sluggish rise) showed the highest loss rate. This pattern likely reflects insufficient luteal support or failing trophoblast function. Low early estradiol has been repeatedly linked to miscarriage. In our cohort, the Trajectory 2 profile essentially replicates the endocrine state seen in many pregnancies that miscarry. Indeed, retrospective studies report that women with persistently low first-trimester estradiol have markedly elevated miscarriage rates. For example, Deng et al. (2022) found significantly lower mean E2 in women who miscarried versus those who did not (13). Likewise, Li et al. confirmed that low E2 values and poor E2 growth rates in the first trimester are strong warning signs of impending loss (26). The clinical importance of Trajectory 2 is underscored by interventional data. Boyle et al. observed that among women with low early pregnancy E2 (below the 50% percentile for gestational age), supplementing with dehydroepiandrosterone (DHEA) (which raises E2) significantly reduced miscarriage rates (27). In their cohort, untreated women with low estradiol had a 45.5% miscarriage rate, whereas those receiving DHEA (which boosted E2) had only a 17.5% loss rate. These findings imply that the high-risk Trajectory 2 does not merely mark failure, but may also be potentially modifiable by improving the hormonal milieu. In practice, our data suggest that patients in the low-E2 trajectory could benefit from closer surveillance or experimental therapies aimed at bolstering luteal steroidogenesis.

### 4.3 Findings from subgroup analyses

In this study, subgroup analyses were performed based on age and the number of previous miscarriages to explore the consistency of the association between estradiol trajectories and the risk of early miscarriage in different populations. The results showed that, regardless of the 20–29 or 30–44 years age groups, the estradiol pattern represented by Trajectory 3 (high level with steady increase) was significantly associated with a reduced risk of early miscarriage. Similarly, in subgroups with one or two previous miscarriages, the association between Trajectory 3 and lower risk of early miscarriage remained stable. These findings suggest that the predictive value of estradiol trajectory for early pregnancy outcomes is generally applicable and is not substantially affected by maternal age or previous history of miscarriage. Notably, no significant associations were observed for the other trajectory groups across different subgroups, further highlighting the unique protective effect of Trajectory 3. However, the sample size in some subgroups was limited, which may have reduced the statistical power.

### 4.4 Limitations

This study has several major limitations. First, as a single-center retrospective study, the sample consisted of outpatients from the Reproductive Center of the Department of Reproductive Medicine, Lanzhou University Second Hospital, which may have introduced selection bias, and the results have limited generalizability and may not be representative of a broader population. Second, although the inclusion and exclusion criteria were relatively strict, the clinical data relied on extraction from hospital electronic medical records, which may have resulted in incomplete information or inaccurate records. In addition, some confounding factors [such as lifestyle (28), smoking (28), diet (29), stress levels (30), vitamin D levels (28),and environmental exposures (31)] were not fully controlled in this study, which may have affected the relationship between estradiol trajectories and pregnancy outcomes. Third, since all participants conceived naturally and patients undergoing assisted reproductive technologies (such as IVF-ET) were not included, the findings may not be directly applicable to those populations. Fourth, the trajectory grouping resulted in a significant imbalance in sample sizes among the four groups. Future research should therefore employ a multi-center, large-sample prospective design to increase the sample size within each group. Fifth, there were individual differences in the number and timing of estradiol measurements, which may have influenced the accuracy of trajectory classification. While batch-level E2 intra- and inter-assay CVs were unavailable, which may constrain a completeassessment of analvtical precision, the standardized Dxl 800 procedures and the physiologicallyplausible, directionally consistent links between E2 trajectories and early pregnancy outcomes offersome reassurance about result reliability. Finally, this study describes the association between E2 trajectories and miscarriage risk but lacks synchronous data on other biological markers (such as progesterone or placental growth factors), thus failing to clarify the specific biological pathways underlying this association. Future studies should use multicenter, large-sample, prospective cohort designs to further validate these findings and explore additional factors that may contribute to dynamic changes in estradiol during early pregnancy.

### 4.5 Conclusion

In summary, this study identified distinct estradiol trajectory patterns during early pregnancy and demonstrated that a high and steadily increasing estradiol level is associated with a significantly lower risk of early miscarriage. These findings highlight the potential value of monitoring estradiol dynamics for early pregnancy risk assessment. Further multicenter, prospective studies are needed to validate these results and explore the underlying mechanisms of estradiol’s role in pregnancy outcomes.

## Data Availability

The original contributions presented in the study are included in the article/**Supplementary Material**. Further inquiries can be directed to the corresponding author.
