# Supplementary material for: Estradiol trajectories and early pregnancy loss: a retrospective study
Source: Front Endocrinol (Lausanne). 2025 Nov 17;16:1657453. doi: 10.3389/fendo.2025.1657453 (PMC12665525; doi:10.3389/fendo.2025.1657453)
Supplement: Supplementary file 1 [file Table1.docx]

**Table S1.** Group-based trajectory modeling results of the model fitting process.

| **No. of latent classes** | **Polynomial degree** | **AIC**^*^ | **BIC**^*^ | **Patients Per class (%)^**^** | **Mean posterior probabilities^***^** |
| --- | --- | --- | --- | --- | --- |
| 1 | Linear | 60947.76 | 60973.36 | NA | NA |
|  | Quadratic | 60901.72 | 60931.6 | NA | NA |
|  | Cubic | 60903.64 | 60937.78 | NA | NA |
| 2 | Linear | 60635.29 | 60677.96 | 85.20/14.80 | 0.94/0.90 |
|  | Quadratic | 60506.50 | 60557.71 | 87.47/12.52 | 0.97/0.92 |
|  | Cubic | 60504.63 | 60564.37 | 87.48/12.52 | 0.97/0.92 |
| 3 | Linear | 60549.59 | 60609.33 | 45.16/11.76/43.07 | 0.80/0.87/0.85 |
|  | Quadratic | 60377.21 | 60449.75 | 67.55/20.87/11.57 | 0.80/0.97/0.84 |
|  | Cubic | 60353.15 | 60438.49 | 6.83/84.06/9.11 | 0.80/0.97/0.84 |
| 4 | Linear | 60536.52 | 60613.33 | 43.26/42.50/1.14/13.09 | 0.77/0.81/0.85/0.88 |
|  | Quadratic | 60247.14 | 60341.02 | 44.78/41.17/4.93/9.11 | 0.82/0.92/0.86/0.87 |
|  | Cubic | 60098.87 | 60209.82 | 6.64/69.07/16.70/7.59 | 0.82/0.92/0.86/0.87 |
| 5 | Linear | 60540.16 | 60634.04 | 45.73/23.91/22.58/1.14/6.64 | 0.81/0.71/0.60/0.84/0.84 |
|  | Quadratic | 60323.67 | 60323.67 | 23.91/44.78/19.17/3.42/8.73 | 0.87/0.84/0.91/0.86/0.86 |
|  | Cubic | 60045.60 | 60182.15 | 0.95/6.07/66.41/17.65/8.92 | 0.87/0.84/0.91/0.86/0.86 |

**Abbreviations**:AIC, akaike’s information criterion; BIC, bayesian information criteria.

*A lower absolute value suggests a better model fit

**No less than 5% of total count in a class

***A higher value is better (preferably > 0.7 in a class)

**Table S2.** Parameter estimates for the best-fitting 4-class cubic group-based trajectory modeling fitted to the estradiol data.

| **Trajectory group** | **Probability of group membership** | **Parameter** | **Estimate** | **SE** | ***P*-value** |
| --- | --- | --- | --- | --- | --- |
| 1 | 6.64% | Intercept | 4479.29 | 1441.77 | 0.002 |
|  |  | Linear | -1203.98 | 614.17 | 0.049 |
|  |  | Quadratic | 99.71 | 86.40 | 0.248 |
|  |  | Cubic | 0.48 | 3.88 | 0.902 |
| 2 | 69.07% | Intercept | -188.17 | 186.55 | 0.313 |
|  |  | Linear | 159.36 | 81.69 | 0.051 |
|  |  | Quadratic | -17.71 | 11.54 | 0.125 |
|  |  | Cubic | 1.21 | 0.51 | 0.019 |
| 3 | 16.70% | Intercept | 4159.72 | 478.16 | <0.001 |
|  |  | Linear | -2089.48 | 226.16 | <0.001 |
|  |  | Quadratic | 345.65 | 34.20 | <0.001 |
|  |  | Cubic | -15.70 | 1.61 | <0.001 |
| 4 | 7.59% | Intercept | -7087.38 | 753.77 | <0.001 |
|  |  | Linear | 3350.36 | 316.30 | <0.001 |
|  |  | Quadratic | -431.29 | 43.58 | <0.001 |
|  |  | Cubic | 18.09 | 1.96 | <0.001 |

**Abbreviations**: SE, standard error of parameter estimate.

**Table S3.** Stratified analysis by age group and the number of previous pregnancy loss.

| **Subgroups** |  | **n** | **OR (95% CI)** | ***P* value** | ***P* for interaction** |
| --- | --- | --- | --- | --- | --- |
| Age,years | Trajectory |  |  |  | 0.100 |
| 20-29 | Trajectory 2 | 132 | ref | - |  |
|  | Trajectory 1 | 10 | 0.15 (0.01,0.89) | 0.080 |  |
|  | Trajectory 3 | 37 | 0.11 (0.02,0.35) | **0.001** |  |
|  | Trajectory 4 | 19 | 0.50 (0.15,1.51) | 0.236 |  |
| 30-44 | Trajectory 2 | 232 | ref | - |  |
|  | Trajectory 1 | 25 | 1.23 (0.51,3.00) | 0.637 |  |
|  | Trajectory 3 | 51 | 0.35 (0.15,0.72) | **0.006** |  |
|  | Trajectory 4 | 21 | 0.39 (0.12,1.11) | 0.095 |  |
| Number of previous pregnancy loss |  |  |  |  | 0.165 |
| 1 | Trajectory 2 | 141 | ref | - |  |
|  | Trajectory 1 | 10 | 0.54 (0.10,2.26) | 0.431 |  |
|  | Trajectory 3 | 37 | 0.17 (0.05,0.47) | **0.002** |  |
|  | Trajectory 4 | 11 | 0.86 (0.21,3.19) | 0.823 |  |
| 2 | Trajectory 2 | 140 | ref | - |  |
|  | Trajectory 1 | 14 | 0.75 (0.21,2.43) | 0.638 |  |
|  | Trajectory 3 | 35 | 0.23 (0.06,0.67) | **0.013** |  |
|  | Trajectory 4 | 16 | 0.29 (0.04,1.18) | 0.127 |  |
| ≥3 | Trajectory 2 | 83 | ref | - |  |
|  | Trajectory 1 | 11 | 0.96 (0.25,3.78) | 0.954 |  |
|  | Trajectory 3 | 16 | 0.39 (0.11,1.24) | 0.119 |  |
|  | Trajectory 4 | 13 | 0.51 (0.14,1.78) | 0.299 |  |

**Abbreviations:** OR, odds radio; CI, confidence interval.
